# Conjugation Inhibitors Effectively Prevent Plasmid Transmission in Natural Environments

**DOI:** 10.1128/mBio.01277-21

**Published:** 2021-08-24

**Authors:** Carolina Palencia-Gándara, María Getino, Gabriel Moyano, Santiago Redondo, Raúl Fernández-López, Bruno González-Zorn, Fernando de la Cruz

**Affiliations:** a Institute of Biomedicine and Biotechnology of Cantabria (IBBTEC), University of Cantabria. Santander, Spain; b Department of Animal Health, Veterinary Faculty, Complutense University of Madrid. Madrid, Spain; c Centro de Vigilancia Sanitaria Veterinaria (VISAVET), Veterinary Faculty, Complutense University of Madrid. Madrid, Spain; Emory University

**Keywords:** *Escherichia coli*, bacterial conjugation, antibiotic resistance, conjugation inhibitor, microcosm, gut, zebrafish, mouse, conjugation, plasmids

## Abstract

Plasmid conjugation is a major route for the spread of antibiotic resistance genes. Inhibiting conjugation has been proposed as a feasible strategy to stop or delay the propagation of antibiotic resistance genes. Several compounds have been shown to be conjugation inhibitors *in vitro*, specifically targeting the plasmid horizontal transfer machinery. However, the *in vivo* efficiency and the applicability of these compounds to clinical and environmental settings remained untested. Here we show that the synthetic fatty acid 2-hexadecynoic acid (2-HDA), when used as a fish food supplement, lowers the conjugation frequency of model plasmids up to 10-fold in controlled water microcosms. When added to the food for mice, 2-HDA diminished the conjugation efficiency 50-fold in controlled plasmid transfer assays carried out in the mouse gut. These results demonstrate the *in vivo* efficiency of conjugation inhibitors, paving the way for their potential application in clinical and environmental settings.

## OBSERVATION

The spread of antibiotic resistance genes (ARGs) among bacterial populations is one of the major threats in global health care. According to existing estimates, approximately 700,000 people die annually because of infections caused by resistant bacteria (1, 2), a figure likely to increase, as the number of strains resistant to various antibiotics is on the rise in a wide range of pathogenic species (3). ARGs may emerge as vertically inherited chromosomal mutations or may be encoded by mobile genetic elements (MGEs) able to spread both vertically and horizontally among bacteria. Among MGEs, conjugative plasmids are of special relevance in the spread of ARGs (4). Conjugative plasmids exhibit a wide host range and thus are able to shuttle ARGs between different genera, orders, and even phyla (5). Moreover, when they invade a new host, they trigger the SOS response, driving the swapping of integron cassettes and promoting the generation of multiresistant strains (6). Preventing plasmid conjugation is therefore a key step in curtailing the propagation of ARGs, and a variety of strategies have been proposed (7, 8). Conjugation inhibitors (COINs) are compounds that inhibit plasmid transfer by affecting the conjugative machinery. Unsaturated fatty acids are COINs blocking the transfer of various groups of plasmids *in vitro* ([Bibr B9][Bibr B10][Bibr B11]). However, their *in vivo* efficiency remained questioned for two major reasons: they require relatively high concentrations to efficiently prevent conjugation, and they can be metabolized by cells as an energy source. On the other hand, synthetic derivatives such as 2-hexadecynoic acid (2-HDA) (C_16_H_28_O_2_) were shown to be more chemically and biologically stable (9). 2-HDA is a potent COIN, able to block the plasmid secretion machinery at low concentrations (12) and prevent conjugation in a variety of plasmids and host species (9), yet its *in vivo* efficiency was not assessed.

We thus decided to test the ability of 2-HDA to block conjugation in two *in vivo* models: a freshwater microcosm and the mouse gut. Water environments are considered hot spots for the propagation of antibiotic resistance, due to the selective pressure exerted by residual antibiotic concentrations present in water effluents (13, 14). The gut is another important niche in the transmission of antibiotic resistance. The gut microbiota acts as a reservoir of ARGs that may be transferred to pathogens (15), and plasmid conjugation has been demonstrated in murine models (16–18) and in the fish gastrointestinal tract (19).

Conjugation experiments were carried out between spontaneous nalidixic (Nx)- and rifampicin (Rif)-resistant derivatives of Escherichia coli MDS42 (20). Conjugation frequencies were determined by plating in appropriate selective antibiotics (21). The water microcosms consisted of three 36-liter aquaria, kept at 28°C and equipped with continuous water flow systems and appropriate filters for water homeostasis, as described in Text S1 in the supplemental material. Aquaria were inhabited by three fish species, Hypostomus plecostomus (suckermouth catfish), Corydoras paleatus (peppered cory catfish), and Danio rerio (zebrafish). Additionally, the microcosms included two species of grass, Vallisneria gigantea (eelgrass) and Lemna minor (common duckweed), and spontaneously acquired invertebrates. We used 16S metataxonomic analyses to characterize the native bacterial population of the aquarium and search for possible species (*Enterobacteriaceae* or other E. coli strains) that could act as conjugation recipients for our experiments (see Fig. S1 in the supplemental material). As described in the supplemental material, neither native aquarium strains nor wild-type E. coli isolated from other aquatic environments (Table S1) behaved better than laboratory strains (Fig. S2 and S3). We thus selected E. coli MDS42 and MDS52 as our recipient strains and checked that 2-HDA did not inhibit or delay bacterial growth (Fig. S4).

10.1128/mBio.01277-21.1 TEXT S1 Supplemental Materials and Methods and supplemental Results on the characterization of the water microcosms. Download Text S1, DOCX file, 0.02 MB. Copyright © 2021 Palencia-Gándara et al. 2021 Palencia-Gándara et al. https://creativecommons.org/licenses/by/4.0/ This content is distributed under the terms of the Creative Commons Attribution 4.0 International license.

10.1128/mBio.01277-21.2 TABLE S1 Escherichia coli strains isolated from river water. Strains were yielded by Jorge Blanco’s laboratory collection (University of Santiago de Compostela). In the table appears the strain number, molecular markers, and phylogenetic group. Download Table S1, DOCX file, 0.07 MB. Copyright © 2021 Palencia-Gándara et al. 2021 Palencia-Gándara et al. https://creativecommons.org/licenses/by/4.0/ This content is distributed under the terms of the Creative Commons Attribution 4.0 International license.

10.1128/mBio.01277-21.3 FIG S1 Freshwater microcosm characterization. (A) Relative abundance of different bacterial phyla in the aquaria. (B) Relative abundance of different bacterial orders in the aquaria. *Enterobacteriales* abundance in each sample is indicated on the bars. Metagenomic DNA obtained from samples of different parts of the aquaria (soil, benthos, plankton, and filters) was extracted, and 16S rDNA was amplified. 16S rDNA sequencing was performed using MiSeq technology, and the resulting reads were analyzed using QIIME2 software to generate an abundance estimation of the different groups of bacteria present in freshwater microcosms. Download FIG S1, TIF file, 1.0 MB. Copyright © 2021 Palencia-Gándara et al. 2021 Palencia-Gándara et al. https://creativecommons.org/licenses/by/4.0/ This content is distributed under the terms of the Creative Commons Attribution 4.0 International license.

10.1128/mBio.01277-21.4 FIG S2 Survival of E. coli MDS52 RifR in aquarium water (AqW) subjected to different treatments. E. coli MDS52 was grown in different situations: aquarium water, filtered aquarium water, boiled aquarium water, and filtered Milli-Q water. A total of 10^5^ to 10^6^ cells were inoculated in water and incubated at 30°C for 96 h. Samples (100-μl sampels) were taken at 0, 2, 24, 48, 72, and 96 h, and dilutions were plated in triplicate on LB agar plates supplemented with the appropriate antibiotics to measure the number of surviving bacteria. Each point shows the mean ± SD of four independent experiments. (A) Conjugation kinetics of the IncF plasmid pOX38 in filtered aquarium water amended with different concentrations of LB broth (0, 1, 3, and 10%). Each point represents the mean ± SD of six independent experiments. (B) Growth kinetics of donor and recipient E. coli MDS52 during the conjugation experiments. Each point indicates the mean ± SD of the total. Download FIG S2, TIF file, 0.3 MB. Copyright © 2021 Palencia-Gándara et al. 2021 Palencia-Gándara et al. https://creativecommons.org/licenses/by/4.0/ This content is distributed under the terms of the Creative Commons Attribution 4.0 International license.

10.1128/mBio.01277-21.5 FIG S3 Environmental E. coli survival in the aquatic microcosm. Equal volumes of 14 strains isolated from river water were mixed and grown overnight at 30°C in aquarium water amended with 10% LB for adaptation. The culture was washed twice, and approximately 10^4^ to 10^5^ cells were inoculated in a flask with aquarium water and sand on the bottom and incubated at 30°C. Samples from water were taken at different time points, while sand samples were taken at the final time. Results shown correspond with one representative experiment. Download FIG S3, TIF file, 0.2 MB. Copyright © 2021 Palencia-Gándara et al. 2021 Palencia-Gándara et al. https://creativecommons.org/licenses/by/4.0/ This content is distributed under the terms of the Creative Commons Attribution 4.0 International license.

10.1128/mBio.01277-21.6 FIG S4 2-HDA effect on E. coli MDS42 growth. Bacterial growth curves were obtained using a Spark 10M plate reader (TECAN) with a 600-nm filter for 96-well microtiter plates. Single colonies were grown in 10-ml flasks at 120 rpm and 37°C overnight. Cultures were diluted 1:1000 in fresh LB medium, and 150 μl of the dilution was transferred to a 96-well plate and incubated inside the plate reader using a humidity cassette (TECAN) to avoid evaporation. *A*_600_ was measured every 5 min. Data were processed using the Python libraries Numpy and Pandas and represented with the Python library Seaborn. Download FIG S4, TIF file, 0.2 MB. Copyright © 2021 Palencia-Gándara et al. 2021 Palencia-Gándara et al. https://creativecommons.org/licenses/by/4.0/ This content is distributed under the terms of the Creative Commons Attribution 4.0 International license.

**FIG 1:**
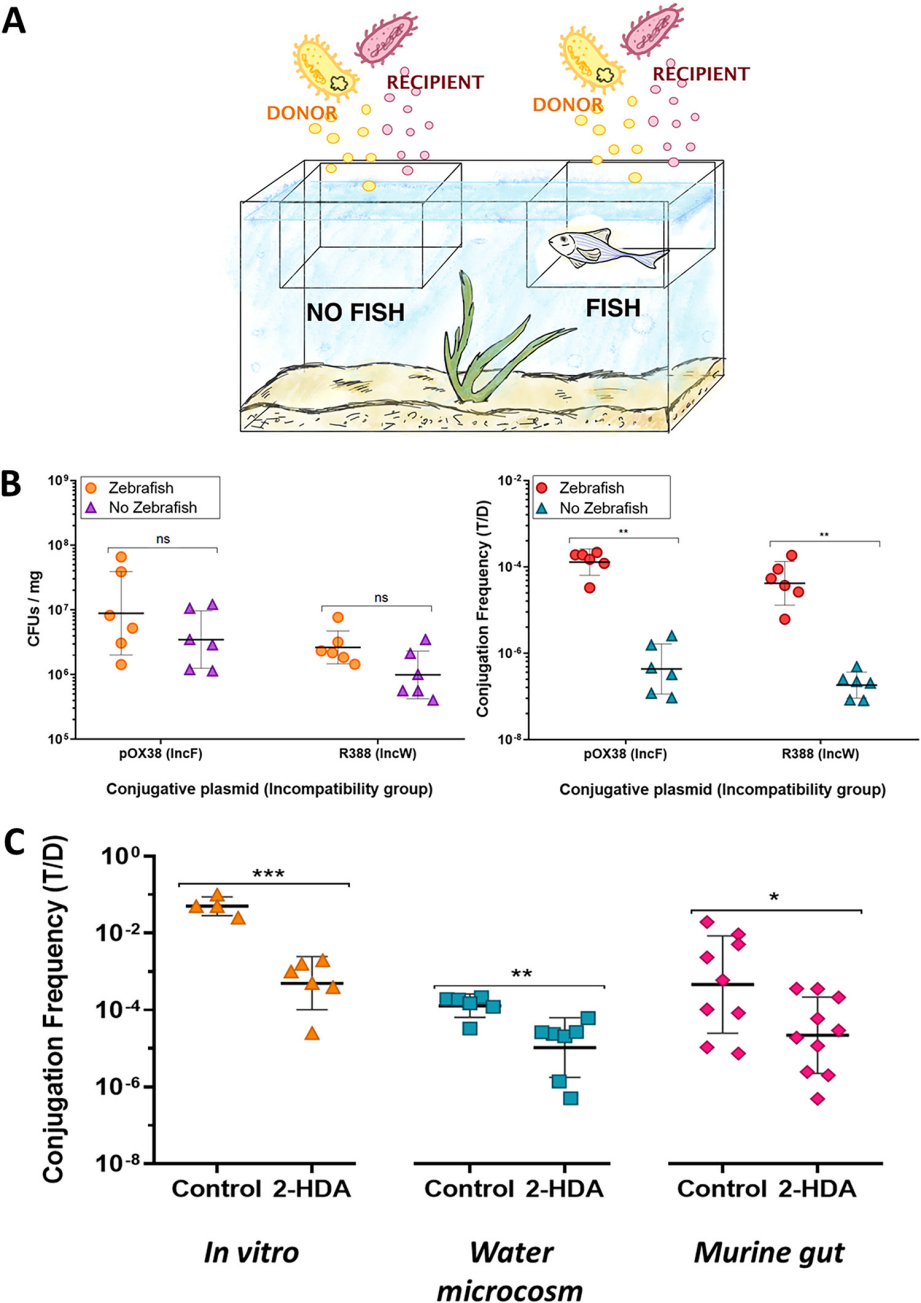
(A) Scheme depicting the administration strategy in water microcosms. Protocol specifications are shown in Text S1 in the supplemental material. (B). E. coli MDS42 survival (left) and conjugation rate (right) in the water microcosms. On the *x* axis are indicated the plasmids carried by the donor bacteria. Each symbol represents the value for an independent experiment. The horizontal bars show the experimental group average. Statistical significance of the average differences, as inferred from a *t* test are shown by asterisks (**, *P* < 0.01; ns, not significant). (C) Effect of 2-HDA administration on the CF of plasmid pOX-38 in different settings. On the *x* axis are indicated the experimental and control groups, while on the *y* axis is the CF expressed as number of transconjugants (T) per donor bacteria (D). Each symbol represents the value for an independent experiment. The horizontal bars show the experimental group average. Statistical significance of the average differences, as inferred from a *t* test are shown by asterisks (*, *P* < 0.05; **, *P* < 0.01).

As shown in Fig. 1A, the conjugation experiments were performed by soaking fish food pellets with donor and recipient bacterial cultures, thus obtaining donor and recipient pellets. These pellets were then added as food to the fish inside a breeding box, installed into the fish tank, to facilitate feeding and the collection of fecal samples. Experiments were performed with and without fishes inside the breeding box, as controls. As shown in Fig. 1B, after 24 h, we retrieved more than 10^6^ CFU per mg of food, when inoculation was performed in empty breeding boxes. When a fish was present in the breeding box, the number of viable counts reached 10^7^ CFU per mg of fecal matter. The results thus demonstrated that the inoculation procedure achieved sufficient cell densities and that bacterial transit through the fish allowed the E. coli viable counts to increase. We calculated conjugation frequencies (CF) using two model plasmids, pOX38, from the PTU-F_E_ group and R388 from the PTU-W group. Results are shown in Fig. 1B. In the absence of fish, conjugation occurred at low frequencies of approximately 10^−6^ transconjugants (T) per donor (D) cell. However, when fishes were present, the CF reached 10^−4^ T/D. These results indicated that transit through the fish gut increased the conjugation frequency of the plasmids.

We then assessed the effect of 2-HDA on the CF. When conjugation was performed *in vitro*, in a growth medium composed of filtered tank water enriched with 10% LB, the CF of plasmid pOX38 reached more than 10^−2^ T/D. This figure was reduced 50-fold when a final concentration of 100 μg/ml 2-HDA was added to the medium. Similarly, when 2-HDA was added to the food at a concentration of 1.6 μg/mg and fed to fish, the CF measured in the fecal pellets decreased >10-fold (Fig. 1C). These results showed that administration of 2-HDA in the food efficiently decreased the ability of plasmids to propagate through conjugation, without inhibiting the bacterial growth rate (Fig. S4).

In order to test whether these results were contingent on the model system, we repeated the experiment in a second setting: the mouse gut. For this purpose, C57BL/6 mice were inoculated with donors and recipients through oral gavage, and the presence of transconjugants in the fecal pellets was evaluated 24 h later, as detailed in Text S1. The presence of confounding Nx^r^ or Rif^r^ E. coli strains in the mice microbiota was ruled out prior to the experiment, as described in Text S1. A dose of 10^9^ cells of donor and recipient bacteria were inoculated to the mice, and supplemented with 100 μg of 2-HDA in the case of the experimental group. When donors and transconjugants from fecal pellets were enumerated, a 10-fold decrease in the CF was observed for the group treated with the COIN. The results thus demonstrated that 2-HDA achieved nearly 90% efficiency in blocking plasmid conjugation in both microcosms (freshwater and mouse gut). Although toxicity was not specifically tested in the experimental setup, no apparent toxic effects were observed in either the fish or the mice during the course of the experiments. Further research is required to better assess potential 2-HDA side effects, as well as determining its impact on the resident microbiota. Altogether, results shown here demonstrate that administration of a prototype COIN into clinical and environmental settings is a feasible strategy to prevent the transmission of antibiotic resistance. Because the persistence of a conjugative plasmid is a delicate balance between the ability to propagate and the burden imposed on the host, a 10-fold decrease in the CF may lead the plasmid to extinction, as demonstrated by previous results (9). Further research is thus required to determine whether COINS can be used *in vivo*, not only to block propagation but also to purge the microbiota from ARG-containing plasmids. This would open the possibility of using COINS as adjuvants to antibiotic treatment in order to prevent the rise and persistence of antibiotic-resistant bacteria.
