# Supplementary material for: Conjugation Inhibitors Effectively Prevent Plasmid Transmission in Natural Environments
Source: mBio. 2021 Aug 24;12(4):e01277-21. doi: 10.1128/mBio.01277-21 (PMC8406284; doi:10.1128/mBio.01277-21)
Supplement: TEXT S1 [file mbio.01277-21-s0001.docx]

**SUPPLEMENTARY INFORMATION**

**1.-Supplementary Materials & Methods**

**Microcosms setting up and characterization**

Freshwater microcosms were installed in three 36 l aquaria, kept at 28-30 ^o^C and equipped with a continuous water flow system and the appropriate filters for system homeostasis. Day-night periods were 8 h light - 16 h darkness. Aquaria were inhabited by three fish species: *Hypostomus plecostomus* (Suckermouth catfish), *Corydoras paleatus* (Peppered cory catfish) and *Danio rerio* (zebrafish). Also, aquaria were populated by two species of plants: *Vallisneria gigantea* (eelgrass) and *Lemna minor* (common duckweed). The aquaria spontaneously acquired various invertebrates, such as snails and insects, together with many eukaryotic microorganisms, which were characterized by cloning the 18S rDNA in E. coli using CloneJet PCR Cloning Kit (Thermo Fisher Scientific) and subsequently sequencing the plasmids. Prior to the experiment, we used 16S metataxonomic analyses to evaluate the presence of native *E.coli* strains that may confound conjugation frequencies. The analysis of the tank microbiota, including results for the water (planktonic), sand (benthic and soil), and filters. For metagenomic DNA isolation, samples from the planktonic and benthic sections of the aquarium were taken. Planktonic samples were obtained by filtering aquarium water followed by resuspension of filters in distilled water. Benthic samples were extracted by vortexing superficial aquarium sand in distilled water. Soil samples were extracted by vortexing deep aquarium sand in water. Filter and filter sponge samples were obtained by vortexing in distilled water. In all cases, bacterial suspensions were spun down and metagenomic DNA was isolated using PowerSoil DNA isolation kit (MoBio) in combination with FastPrep24 instrument. DNA quality was checked with a Bioanalyzer 2100 (Agilent technologies). Two consecutive PCR steps, to amplify regions V3-V4 of the bacterial 16S rDNA (Primers recommended by Illumina guideline: 341f 5’-CCTACGGGAGGCAGCAG-3’, 805r 5’-GACTACHVGGGTATCTAATCC-3’) and attach the adaptors for sequencing were performed as part of the library preparation before sequencing. MiSeq technology (Illumina) was used to sequence bacterial samples, generating from 60,000 to 100,000 non-chimeric reads per sample (**Table S1**). V3-V4 primers were cleaned from raw reads with CUTADAPT. ASV amplicon sequence variants were extracted with DADA2 and subsequently classified in taxons with SILVA vers. 132. Metagenomic data were analyzed using QIIME2 software (http://qiime2.org/).

**Bacterial strains and plasmids**

*Escherichia coli* reduced genome strains MDS42 and MDS52 (1) were used as donors and recipients in conjugation experiments. Spontaneous mutants of MDS42 and MDS52 resistant to nalidixic acid 20 μg/ml (Nx^R^) and rifampicin 50 μg/ml (Rif^R^) were used as donors and recipients respectively. Model conjugative plasmids pOX38::*CmR* (IncF1), and R388 (IncW), resistant to chloramphenicol 25 μg/ml (Cm), and trimethoprim 20 μg/ml (Tp) respectively, were used for conjugation experiments. For the experiments, bacteria were grown overnight in Luria Bertani broth (LB) supplemented with the appropriate antibiotics at 37 °C with agitation.

**Survival of *E. coli* in aquarium water**

As part of the microcosm characterization, growth kinetics to assess *E. coli* survival in aquarium water were performed. *E. coli* MDS52-Rif^R^ was grown overnight, cultures were washed twice and cells were resuspended in the corresponding water for the experiments: aquarium water, filtered aquarium water (using Millipore 0.22 μm filters), boiled aquarium water (10 min) and filtered Milli-Q water. A total of 10^5^-10^6^ cells were added to tubes containing 1 ml of water and incubated at 30 ^o^C. Samples were taken at 0, 2, 24, 48, 72 and 96 hours and dilutions were plated on Brilliance *E. coli*/Coliform Selective Agar (Oxoid, France) supplemented with Rif to determine the number of surviving bacteria. When *E. coli* was inoculated directly into the aquarium the population rapidly decayed. Further analyses revealed MDS52 maintained or even increased its numbers in filtered or boiled aquarium water. On the other hand, non-filtered water caused a sharp decrease on viable counts after 48 h (**Figure S2**). Bacterial mortality was not due to maladaptation, but most likely caused by some non-filterable component of the water microcosms (e.g., predatory protozoa), since wild-type *E.coli* strains isolated from aquatic environments behaved no better (**Table S2**, **Figure S3**).

**Conjugation in aquarium water**

For conjugation experiments in filtered water from the aquarium, donor and recipient strains were grown overnight at 37 ^o^C, cultures were washed twice and cells were resuspended in filtered aquarium water. 10^7^ cells from donor and recipient bacteria were added to tubes filled with 2 ml filtered aquarium water amended with different concentrations of LB broth (0, 1, 3 or 10 %), to simulate a polluted environment. Tubes were incubated at 30 °C with gentle agitation (80 rpm) and samples were taken at 0, 2, 4 and 24 h. Dilutions were plated on LB agar supplemented with the appropriate antibiotics to select donors (Nx + Cm), recipients (Rif) and transconjugants (Rif + Cm). Conjugation frequencies were calculated as the ratio of transconjugants per donor cell (T/D). In inhibition experiments, 100 µg/ml of 2-hexadecynoic acid (2-HDA) or the equivalent volume of DMSO (carrier) were added to the tubes.

**Conjugation in zebrafish gut**

Donor and recipient bacteria were grown overnight at 37 ^o^C in LB broth supplemented with the appropriate antibiotics. 10 ml of the saturated cultures (10^9^ cells/ml) were washed once and centrifuged again. Resulting pellets were resuspended in 20 μl of sterile phosphate buffered saline (PBS) (Fisher Scientific, NJ) and 300 mg of granulated fish food were added. The mixtures were homogenized until food was impregnated with bacteria. For inhibition experiments, 500 µg 2-HDA or the equivalent volume of DMSO were added to the bacterial mass before mixing with the food, making a final concentration of 1.6 μg/mg. Pairs of zebrafish were transferred to 1 l breeding boxes filled with aquarium water. Fish were fed with approximately 20 mg of donor-impregnated and recipient-impregnated granules, receiving a total of 64 µg 2-HDA per box (approx. 32 µg per fish). Two hours post-feeding, the bottom surface of the breeding boxes was isolated with a grid that allows the feces to fall but avoids fishes to get to the bottom. After 24 h, feces were collected with a Pasteur pipet, centrifuged, diluted and plated on Brilliance *E. coli*/Coliform Selective Agar (Oxoid, France) supplemented with the appropriate antibiotics to select donors (Nx + Cm), recipients (Rif) and transconjugants (Rif + Cm). Conjugation frequencies were calculated as the transconjugants/donors ratio. Presence of the plasmid in transconjugant cells was confirmed by PCR using the primers pOX38_Fw 5’-GCTGGGCAGCATGGGAGAAC-3’ and pOX38_Rv 5’-CAATCTGATTAGCGTACACATTCTCAATG-3’ (amplicon = 509 bp) or R388_Fw 5’-CATAGGGCGGGCTGCAAGC-3’ and R388_Rv
5’-GTGGCCCTCGCCGATATTCC-3’ (amplicon = 169 bp).

**Mice**

The experimental protocol was approved by Directorate General of Agricultural and livestock of the Community of Madrid. In all the experiments, four-week-old male C57BL/6 mice (Charles River Laboratories, France) were used. The mice were housed in groups of five during a week for acclimatization. Before the inoculation day, mice were housed individually. The presence of bacteria resistant to Nx, Cm and Rif in the fecal microbiota, that may interfere in the experiment, was tested in the naïve mice by collecting all the feces pellets independently from the cage of each individually housed mouse after 24 h. The pellets were suspended in 5 ml of sterile PBS and homogenized during 5 min in a Masticator (IUL Instruments, SP). Then, 100 μl of each sample were plated in triplicate in Brilliance *E. coli*/Coliform Selective Agar with Nx, Rif, or Cm. Mice harbouring resistant bacteria were discarded for the experiments.

**Conjugation in a mouse gut model**

Mice were inoculated by oral gavage with suspended in 100 μl of PBS (Fisher Scientific, NJ) and supplemented with 100 μg of 2-HDA in the case of the experimental group. After 24 h, feces pellets were collected and weighed. The pellets were suspended in 5 ml PBS and homogenized for 5 min in a Masticator (IUL Instruments, Spain). Dilutions of the samples were plated on Brilliance *E. coli*/Coliform Selective Agar supplemented with the appropriate antibiotics to select donor, recipients and transconjugant cells. Conjugation frequencies were calculated as the ratio of transconjugants per donor cell (T/D). To accurately count the number of transconjugants, 20 plates with the corresponding antibiotic were plated with 100 μl of the sample. Presence of the plasmid was confirmed by PCR using the primers pOX38_Fw and pOX38_Rv previously described.

**Statistical analysis**

Comparison of the means between two different conditions was carried out by using t-test tool from GraphPad Prism® (v 7.0.4) biostatistics software (San Diego, CA).

**2.-Supplementary results**

**Aquatic microcosm characterization**

After setting up the aquatic microcosm, a metagenomic analysis was performed. Samples were obtained from water (planktonic), sand (benthic and soil), and filters. In **Figure S1-A** are shown the phyla represented in the different parts of the 3 aquaria set up. Benthic and soil samples were homogeneous among the different aquaria. In benthic samples the most abundant phyla were Proteobacteria and Bacteroidetes, while in soil samples, the phyla Cyanobacteria, Chloroflexi and Verrucomicrobia fractions were also majority. Planktonic samples show the greatest differences among aquaria. Filters were sampled only in one of the aquaria, and both the filter and the sponge showed a similar microbiome, with Proteobacteria being the among the 70-80 % of the OTUs sampled. In general, the class γ-proteobacteria was the most represented in most of the parts. Among γ-proteobacteria, the order β-proteobacteriales (red) was the most abundant in the samples (**Figure S1-B**). The order Enterobacteriales was poorly represented, ranging from 0.01 % in the filter sample to 0,18 % in one of the soil samples (**Figure S1-B**)**.** The Enterobacteriales found in the metagenome correspond to *Plesiomonas shigelloide, Photorhabdus temperate, Escherichia coli* and the Klesiella genera. In an attempt to mimic the conditions in wastewater, we decided to introduce *E. coli* exogenously to colonize the microcosms with a clinically relevant conjugation recipient detected in this type of environments (2). When *E. coli* was inoculated into the aquarium, either in water or sand, the population rapidly decreased and after two days, it completely disappeared (data not shown). To assess the reason for the mortality, laboratory strain *E. coli* MDS52 was added to 1 ml aquarium water with different treatments. Counts of *E. coli* throughout time were drastically reduced in untreated aquarium water (**Figure S2**) and maintained in filtered aquarium water (using Millipore 0.22 µm filters) and filtered Milli-Q water. Only in boiled aquarium water, *E. coli* was able to grow, probably due to the release of nutrients from lysed cells. These results suggest that *E. coli* is efficiently eradicated by some non-filterable components of the aquarium water. Even if we inoculated the aquaria with a mixture of preadapted (grown at 30 ^o^C in aquarium water + LB) *E. coli* strains isolated from rivers, this bacterium seems unable to survive more than two days under the experimental conditions (**Figure S3**).

**3.- Supplementary references**

1. Pósfai G, Plunkett G, Fehér T, Frisch D, Keil GM, Umenhoffer K, Kolisnychenko V, Stahl B, Sharma SS, de Arruda M, Burland V, Harcum SW, Blattner FR. 2006. Emergent properties of reduced-genome Escherichia coli. Science 312:1044–6.

2. Amos GCA, Hawkey PM, Gaze WH, Wellington EM. 2014. Waste water effluent contributes to the dissemination of CTX-M-15 in the natural environment. J Antimicrob Chemother 69:1785–1791.
