# Supplementary material for: Conjugation Inhibitors Effectively Prevent Plasmid Transmission in Natural Environments
Source: mBio. 2021 Aug 24;12(4):e01277-21. doi: 10.1128/mBio.01277-21 (PMC8406284; doi:10.1128/mBio.01277-21)
Supplement: TABLE S1 [file mbio.01277-21-st001.docx]

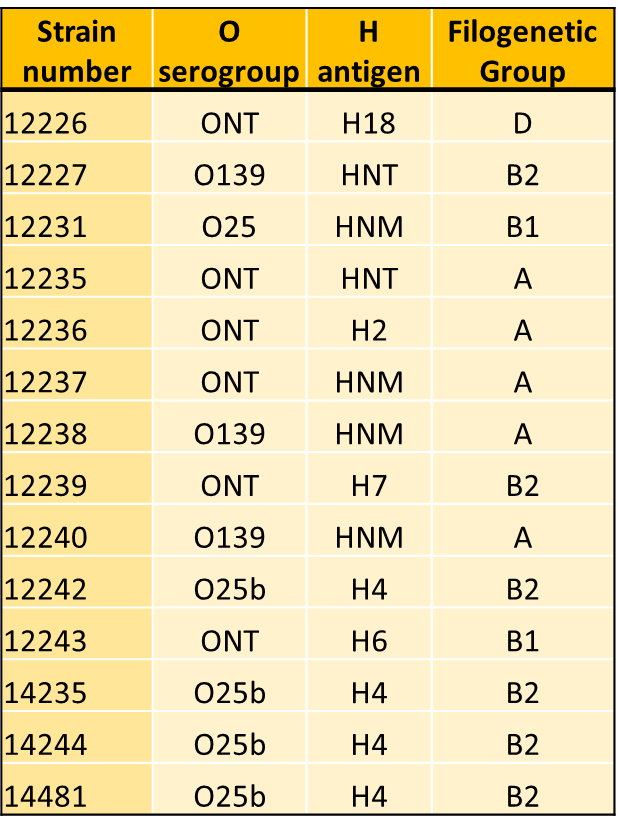
**Table S1. Escherichia coli strains isolated from river water.** Strains were yielded by Jorge Blanco’s laboratory collection (University of Santiago de Compostela). In the table appears the strains number, molecular markers and filogenetic group.
