# Supplementary figures and images for: Conjugation Inhibitors Effectively Prevent Plasmid Transmission in Natural Environments
Source: mBio. 2021 Aug 24;12(4):e01277-21. doi: 10.1128/mBio.01277-21 (PMC8406284; doi:10.1128/mBio.01277-21)

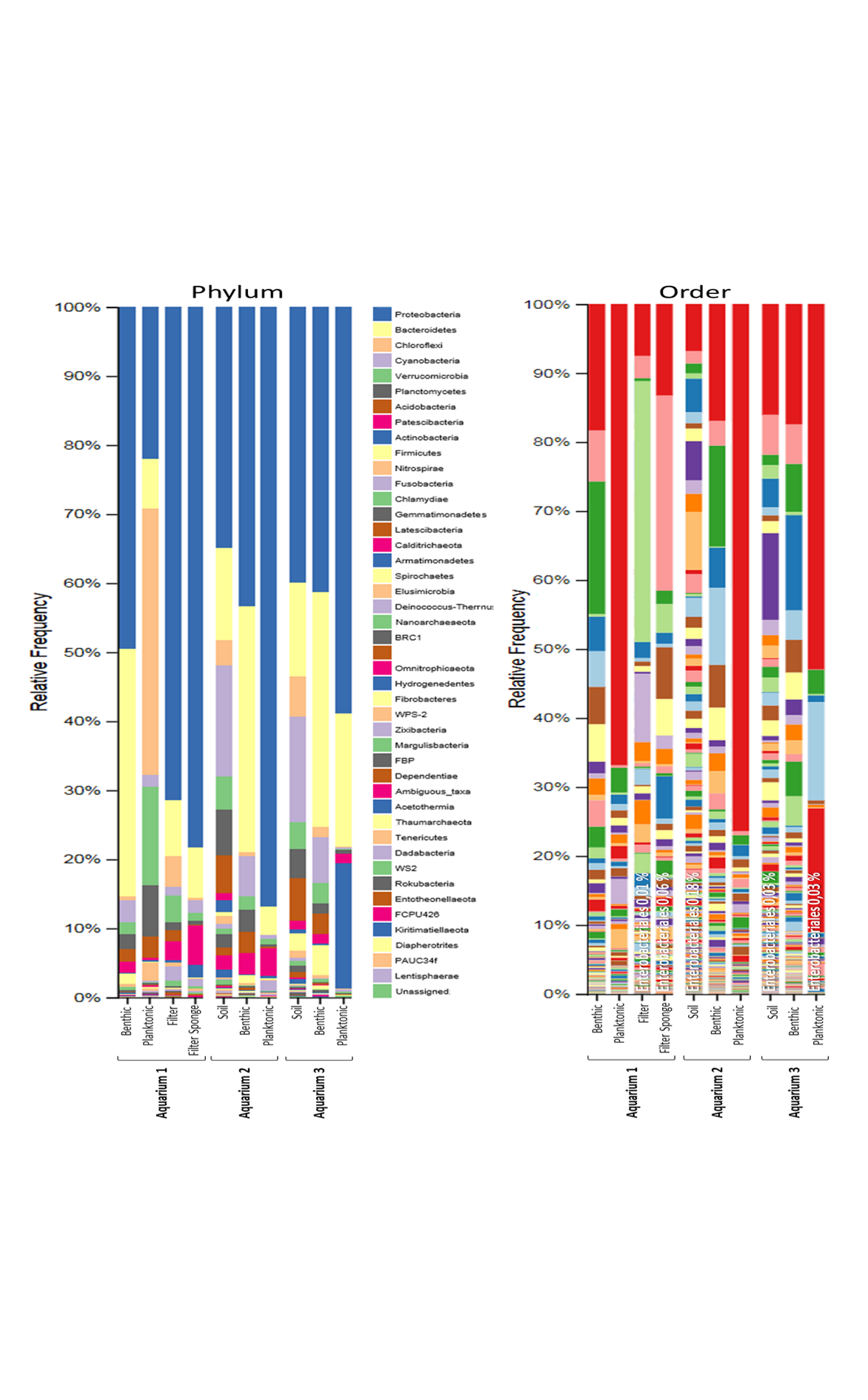

Supplement: FIG S1 [file mbio.01277-21-sf001.tif]

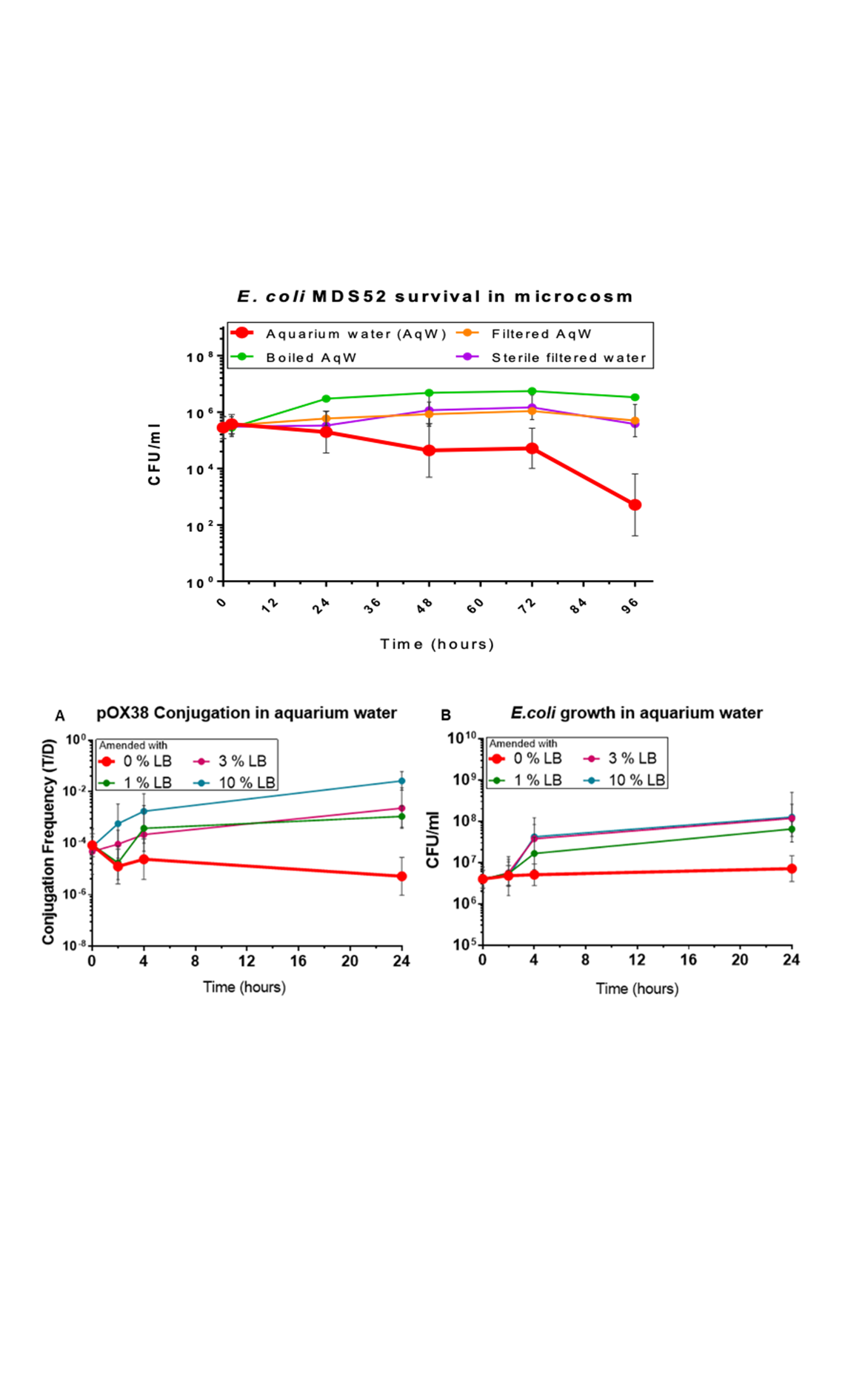

Supplement: FIG S2 [file mbio.01277-21-sf002.tif]

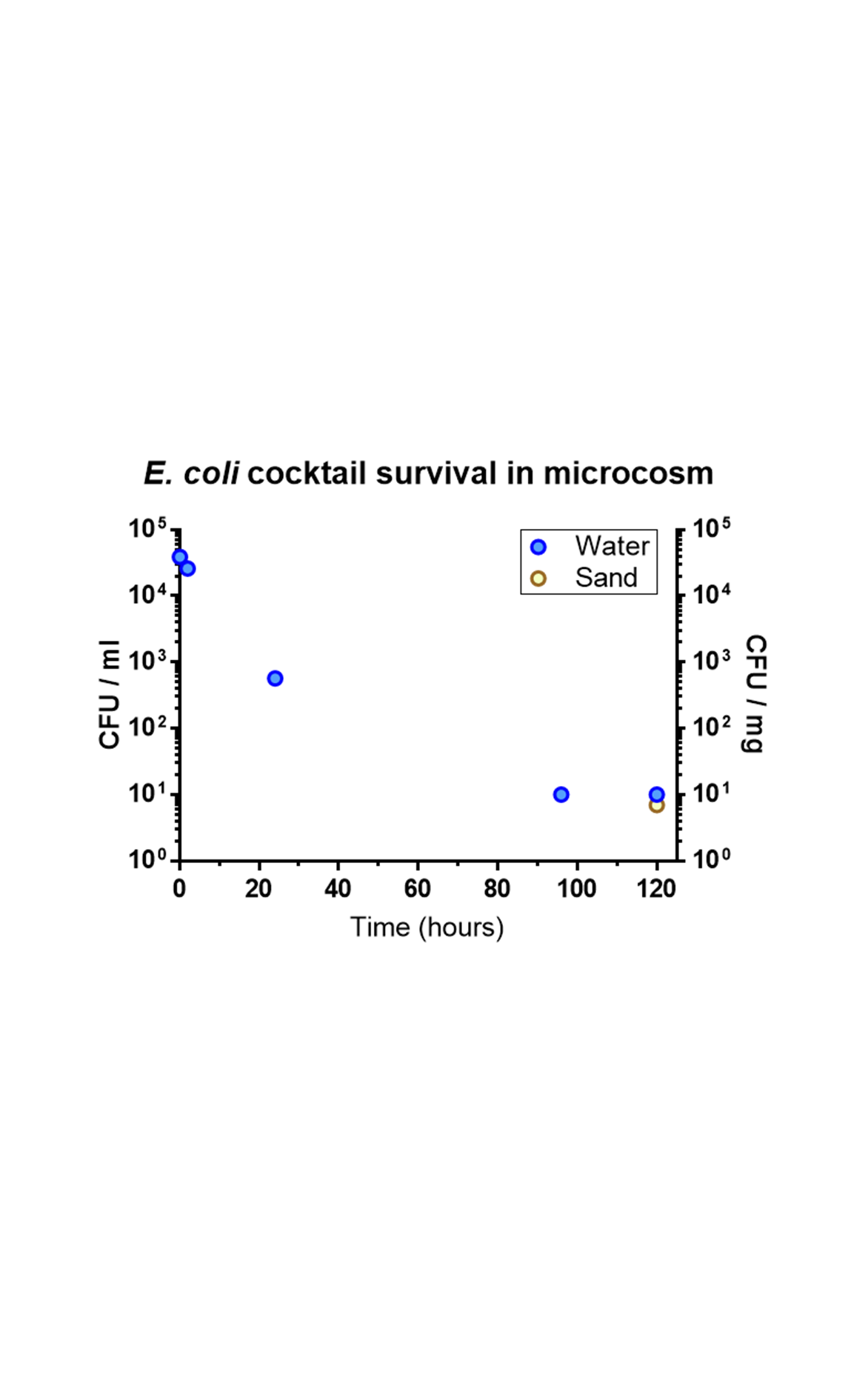

Supplement: FIG S3 [file mbio.01277-21-sf003.tif]

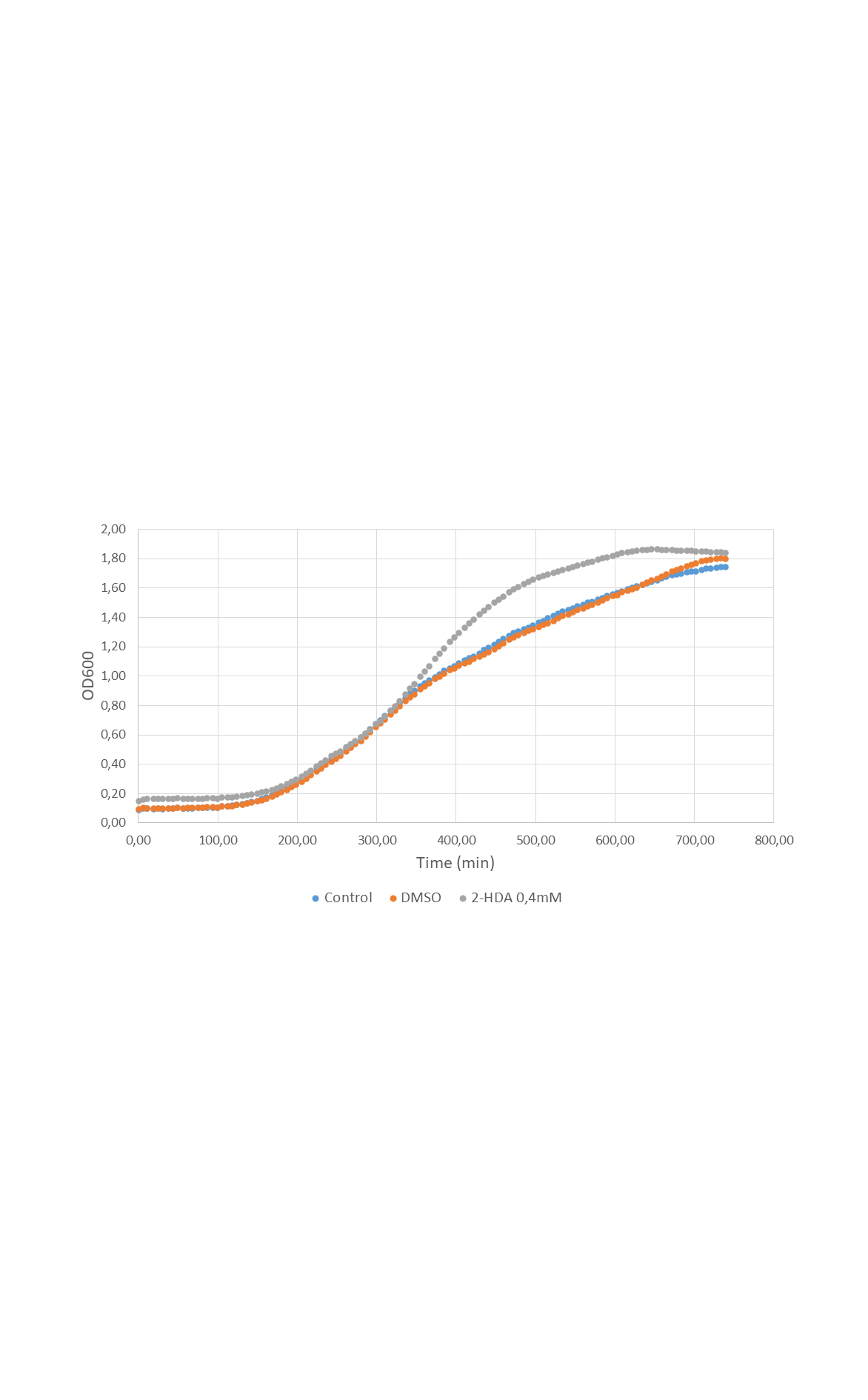

Supplement: FIG S4 [file mbio.01277-21-sf004.tif]
